# An In Silico Patient-Specific Computational Framework for a Tissue-Engineered Pulmonary Valve Using Fluid-Structure Interaction Analysis

**DOI:** 10.1007/s10439-026-04171-0

**Published:** 2026-05-06

**Authors:** Fabrizio Crascì, Tahir Turgut, Maria Bastrom, Deniz Oztruk, Nils Götzen, Martijn Cox, Salvatore Pasta

**Affiliations:** 1 Department of Engineering, Università degli Studi di Palermo, Viale delle Scienze Ed.8, Palermo, Italy; 2 Department of Research, IRCCS, ISMETT, via Tricomi, 5, Palermo, Italy; 3 4RealSim Services BV, Groene Dijk 2B, 3401 NJ IJsselstein, Netherlands; 4 Capvidia, Technologielaan 3, 3001 Leuven, Belgium; 5 Xeltis BV, De Lismortel 31, 5612AR Eindhoven, The Netherlands

**Keywords:** Tissue-engineered heart valves, Fluid-structure interaction analysis, Pulmonary valve, Validation

## Abstract

Tissue-engineered heart valves provide significant promise for pediatric patients by overcoming the shortcomings of existing prostheses, which do not accommodate the patients’ growth. The Xeltis pulmonary valve (XPV) has recently shown promising clinical outcomes for endogenous tissue restoration in pediatric patients with congenital heart diseases. In this context, patient-specific computational modeling may be pivotal in the advancement and clinical application of in situ polymer-based cardiac valves, enhancing design, facilitating regulatory approval, and diminishing dependence on preclinical animal testing. This study seeks to establish a computational framework for incorporating the XPV device into patient-specific models and assessing the device’s biomechanical performance via fully coupled fluid-structure interaction (FSI) simulations. Hemodynamic parameters were predicted and compared with echocardiographic data from the prior Xeltis clinical trial. FSI analyses showed good agreement with clinical assessments, with errors in the device’s transmural pressure gradient ≤ 10%. Simulations further confirmed that XPV maintains favorable hemodynamics and mechanical integrity throughout the cardiac cycle. This computational framework can facilitate design optimization and tailored implantation strategies for innovative tissue-engineered heart valves. Although further validation with extensive clinical data is required to confirm the model’s credibility, this framework provides a foundation for in silico trials to evaluate the long-term restorative performance of the XPV device.

## Introduction

The next generation of tissue-engineered heart valves is highly anticipated in cardiac valve surgery as it promises to address critical unmet clinical requirements for the pediatric population [1]. The approach involves the initial implantation of a biodegradable valve, which is then replaced by the patient’s own tissue during the post-surgical period. The biodegradable valve is generally an electrospun polymer placed in situ as a cell-free, fully synthetic scaffold or as tissue-engineered matrices seeded with cells to facilitate neo-tissue formation and ultimately the development of the heart valve structure. This method should address the limitations of existing standard surgical strategies based on autografts or allografts, which inherently lack the capacity for somatic growth with the patient’s body [2, 3]. Despite the significant promise for developing regenerative valve substitutes, many challenges must be overcome for a large-scale application in clinical practice, including the inherent regeneration properties and regulatory requirements [4].

The Xeltis pulmonary valve (XPV) device is a fully synthetic, bioabsorbable conduit featuring three valve leaflets that has recently demonstrated encouraging clinical outcomes for endogenous tissue restoration in pediatric patients. In a recent study, the XPV device was used in twelve pediatric patients requiring right ventricular outflow tract repair and shown favorable outcomes at twelve months post-implantation [5]. Computational modeling could help the safe and effective clinical translation of tissue-engineered cardiovascular implants by enhancing design and testing while minimizing costs and ethical concerns associated with preclinical animal studies [6]. Additionally, patient data can be incorporated to tailor the computational model for each individual case, thereby predicting the biomechanical response of the candidate medical devices under realistic in vivo conditions. Tough patient-specific computational modeling of tissue-engineered heart valves has been inadequately explored. Fluid-structure interaction (FSI) approaches have been widely applied to investigate the biomechanics of the aortic valve [7]. FSI approaches have also been applied to the pulmonary circulation and pulmonary valve replacement, including patient-specific simulations in Tetralogy of Fallot and right ventricular outflow tract reconstruction [8, 9]. These studies highlighted the capability of FSI frameworks to capture the complex interaction between flow and valve kinematics, providing insight into device performance and surgical planning. In the context of tissue-engineered valves, such approaches are particularly relevant for quantifying biomechanical stimuli that may govern growth and remodeling processes.

This study sought to provide a computational framework for integrating the XPV device into patient-specific models and assess the biomechanical performance of the device using fully coupled FSI analyses. A probabilistic evaluation of the predictive capabilities of the patient-specific XPV model was carried out by comparing key hemodynamic metrics with echocardiographic evaluations reported in the Xeltis clinical trial involving human subjects. Fluid shear stress and maximum principal stress were calculated using the structural and fluid dynamic solver as key parameters for controlling growth and remodeling. The proposed FSI approach enabled a realistic assessment of XPV device performance and is essential for establishing model credibility and developing in silico clinical trials for evaluating the long-term response of the regenerative valve substitutes.

## Methods

### Patient Study Group

Retrospective phase-contrast magnetic resonance imaging (MRI) data of twelve pediatric patients with Tetralogy of Fallot were collected at the ISMETT Hospital. For comparative purposes, the patient age of computational models was matched to that of the pediatric cohort described by Morales et al. [5], who reported a median age of 6 years (range: 3–9 years). Specifically, the study cohort included twelve pediatric patients with a mean age of 6.7 ± 3.4 years, of whom 65% were male. The average body surface area was 1.28 ± 0.36 m^2^. Cardiac function was characterized by a mean left ventricular ejection fraction of 56.0 ± 7.2%, a right ventricular ejection fraction of 46.5 ± 5.3%, and a right ventricular stroke volume of 60.1 ± 28.3 mL.

From the Morales study, the first four patients for each device group were extrapolated to align with the virtual implantation simulations in the present study, with four retrospective patients assigned to each device group accordingly. Moreover, the average transmural pressure gradient across the XPV valve leaflets (14.1 ± 5.0 mmHg) and regurgitation fraction, as reported by Morales and collaborators [5], were used as reference values for validation of the patient-specific XPV model in our cohort.

### XPV Device Modeling and Integration

#### Device Description

**Fig. 1:**
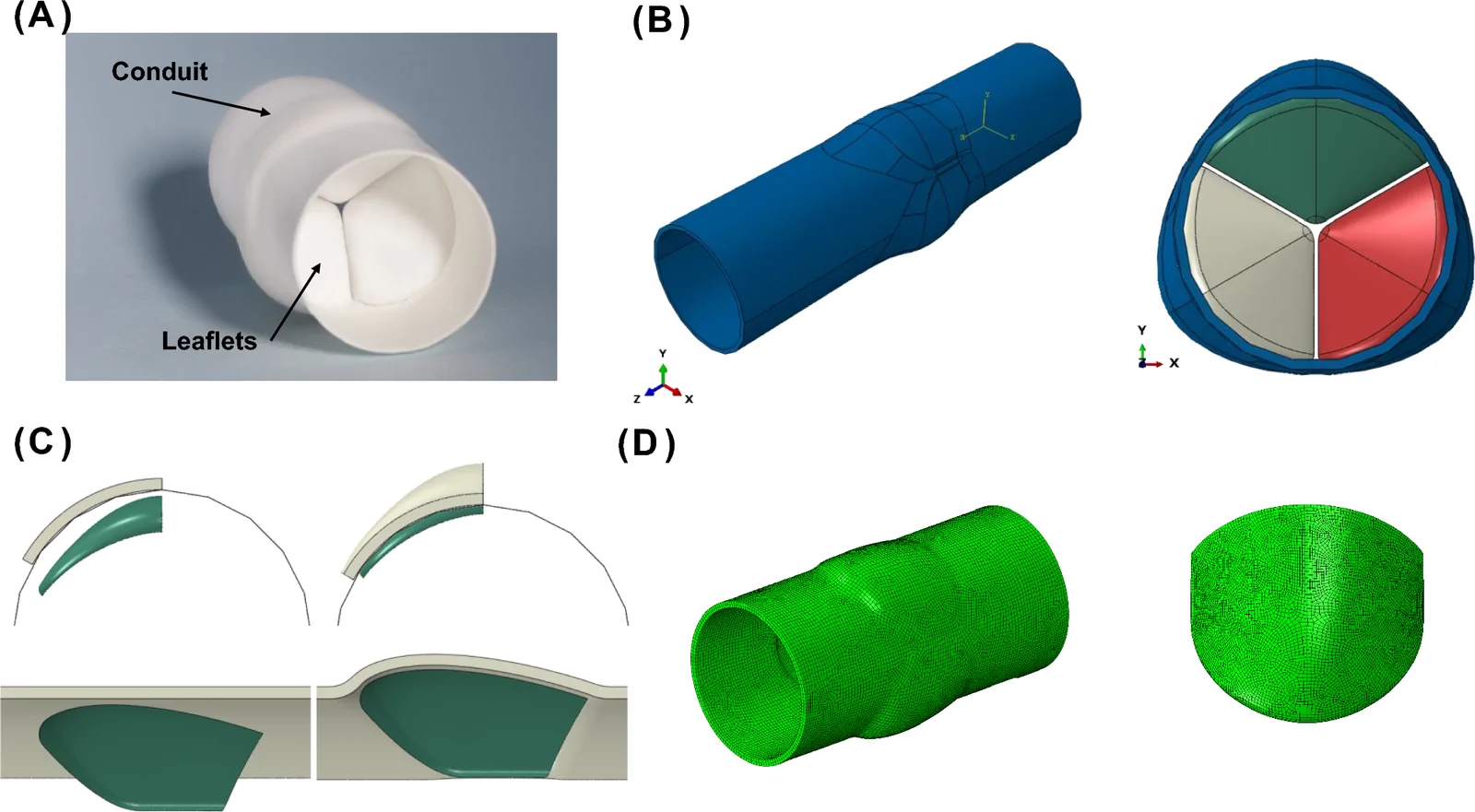
**A** Photography of the XPV device and **B** the CAD model of the conduit with the view of the valve leaflets, **C** steps of the forming simulation used to generate the finite-element model, showing the deformation of the initial cylindrical tube (white) into the final conduit–leaflet configuration (green), including the sinus conformation and leaflet attachment, **D** mesh of the XPV conduit and valve leaflets

The XPV device consists of a cylindrical tube featuring three sinuses and valve leaflets, constructed from biodegradable scaffold material (Fig. 1A). The diameter of the cylindrical tube corresponds to the size of the XPV device. Valve leaflets have a triangular, cap-like morphology, and are nearly closed in the stress-free condition. A micro-CT scan of the 20 mm XPV conduit was performed to obtain the device shape and thickness, followed by reverse engineering to reconstruct the geometry (Fig. 1B).

#### CAD Integration of XPV in Patients Models

**Fig. 2:**
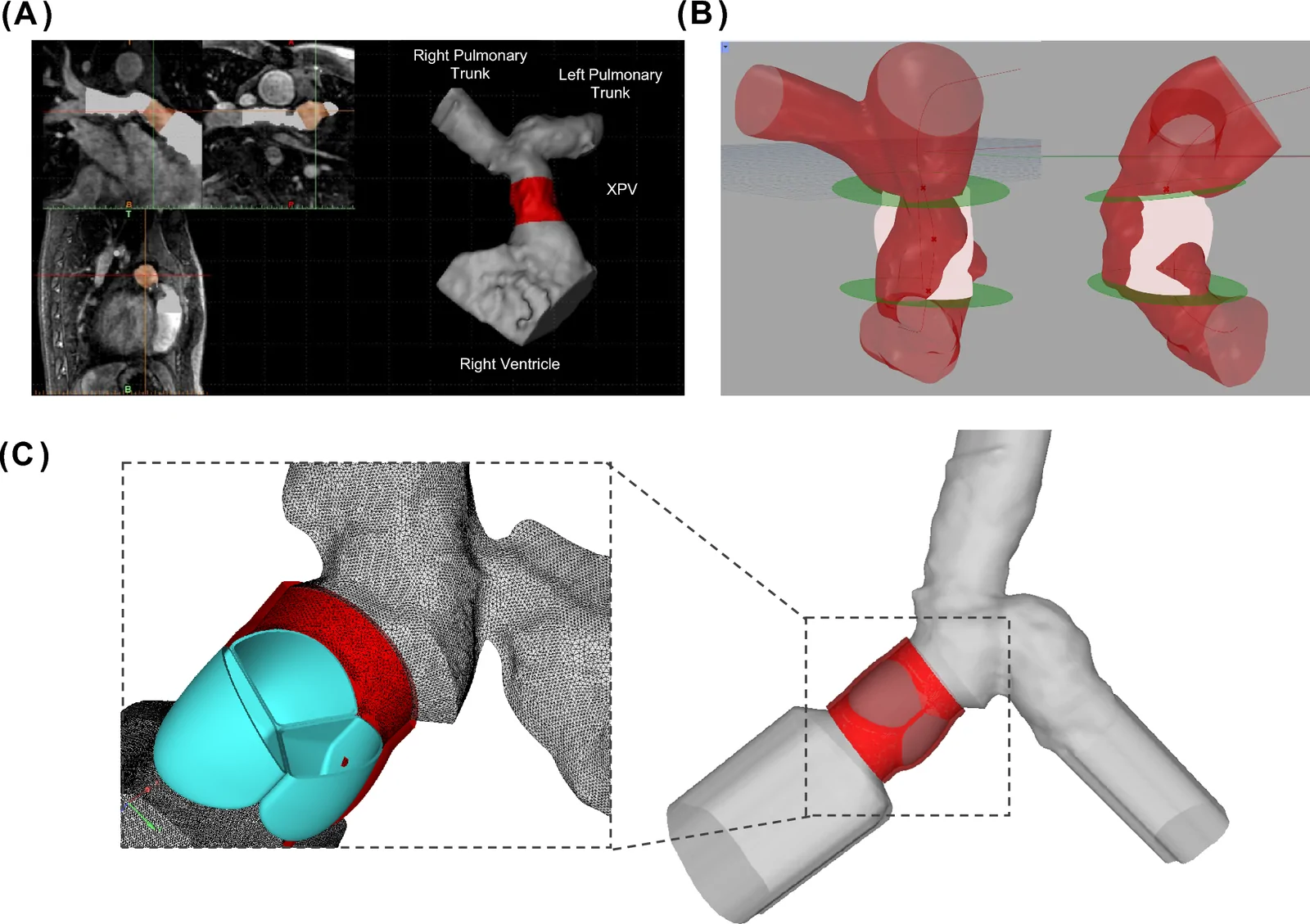
**An** MRI scan of one patient with the region of the XPV device’s intended position highlighted in red color, **B** two views of the CAD modeling framework implemented in Rhino showing the trimming plane (green), the XPV conduit (white), and the pulmonary vessel (red), **C** element mesh of the pulmonary wall together with the integrated XPV model

A 3D modeling framework was built within the Grasshopper application of Rhinoceros software (v.8, McNeel, USA) to integrate the XPV model in patient anatomies and determine the locations of planes required for trimming the XPV device (Fig. 2A and B). The positioning of the XPV device was performed with the support of experienced surgeons and guided by consistent anatomical landmarks to enhance reproducibility across cases; however, this semi-manual procedure may introduce operator-dependent variability. This can potentially affect the predicted hemodynamic outcomes. The XPV device is constructed as a conduit, with an extension sufficiently long to allow modification during surgery based on the length of the patient’s pulmonary vessels. The XPV geometry was imported into Grasshopper and then aligned by rotation and translation to the centerline of the patient’s pulmonary artery. Two planes, positioned proximally and distally to the XPV sinuses, were manually aligned and then employed to modify the device according to the surgeon’s specifications. An input point was used to control the device along the tangent of the vessel centerline to place the XPV device with respect to the height of the pulmonary vessel.

#### Finite-Element Model of XPV

The CAD model of the XPV device was employed to generate ad hoc forming simulations to ensure model symmetry and prevent element mesh distortion (Fig. 1C). The forming simulation employed a finite-element-based quasi-static approach in Abaqus/Standard, in which a stiff cylindrical component was used to transform the initial cylindrical mesh tube into the final XPV conduit geometry. The CAD model of the valve leaflets, which includes both the leaflet belly and sinus regions, was then used to prescribe displacement boundary conditions on the conduit, thereby inducing the sinus conformation and defining the leaflet attachment to the conduit wall. Contact conditions were subsequently applied to ensure proper coupling between the leaflets and the conduit. This procedure was adopted to ensure geometric consistency and mesh quality of the XPV model rather than to replicate a physical forming process. The procedure is illustrated in Fig. 1C, where the initial cylindrical tube (white) is deformed into the final leaflet-sinus configuration (green).

The XPV material was modeled using a novel constitutive relationship characterized by an anisotropic hyperelastic response, stress softening, permanent deformation, and rate dependency [10]. The free-energy function comprises an equilibrium component that accounts for the time-dependent material response and a non-equilibrium component for the viscoelastic distribution. The elastic response is characterized by an anisotropic strain energy function, incorporating discrete orientation to represent fiber direction in the device model. A rigorous numerical verification of mesh size, solver configurations, and code implementation was carried out as part of the Horizon 2020 Project of the European Union, SimInSitu. Mesh convergence was assessed by monitoring the maximum principal stress on the leaflet belly. This numerical activity attempted to keep finite-element errors ≤ 1% by making model input negligible in comparison to errors arising from model assumptions (e.g., materials and boundary conditions). The optimal element mesh topology included solid elements with reduced integration (C3D8R, with size of 0.1 mm) and included three elements in the thickness direction (Fig. 1D). The structural simulations were carried out using Abaqus/Explicit using mass-scaling of 1.0e-6. A contact algorithm with penalty of 0.1 was adopted for the contact among leaflets, with viscous pressure applied on leaflet surfaces to reduce transient effects. The structural model was also validated against in vitro data, including comparisons of effective orifice area and mean transvalvular pressure gradient measured in a dedicated flow loop [11].

### Patient-Specific XPV Model

The pulmonary artery was segmented from the right ventricular outflow tract to the distal pulmonary trunk using semi-automatic thresholding of MRI images with the medical imaging software Mimics (v21, Materialise, Belgium) [12]. Patient-specific geometries were segmented from MRI data using intensity-based thresholding on image gray levels, with thresholds selected within the high-intensity range corresponding to the blood pool and adjusted for each patient and acquisition protocol. The proximal and distal ends were cut perpendicular to the vessel centerline and then extended sixfold to guarantee stable flow at the boundaries. Finally, pulmonary trunk geometries were refined through manual correction. The vessel wall was discretized using triangular shell (S3R) elements with a dimension of 0.15 mm and a thickness of 1.5 mm.

The zero-pressure configuration of the artery was calculated using the method suggested by Krishnan et al. [13], since segmentation was done using in vivo images of children during normal respiration. The pulmonary vessel was assumed to have supra-physiological stiffness (Young’s modulus of 1 × 10^9^ Pa) and was loaded to a diastolic pressure of 8 mmHg. This condition aimed to provide a stress distribution on the vessel wall without causing deformation due to the rigid material properties. The resultant stress distribution is utilized as the initial condition for an additional analysis in which the supra-physiological rigid material is replaced with the actual patient-specific material properties. The pulmonary artery was then deflated from diastolic pressure to 0 mmHg, resulting in the zero-pressure unloaded shape of the arterial wall. Patient-specific material properties were established by an inverse analysis while assuming the arterial wall to be a Neo-Hookean model [14]. The inverse analysis permitted the minimization of the difference between the diastolic-to-systolic strain predicted by simulations and the measurements obtained from MRI scans. The identified Neo-Hookean material parameters representing the global biomechanical response of the pulmonary vessel in the patient study group were C1 = 0.0206 ± 0.0118 MPa and D1 = 3.72 ± 2.95 MPa.

The trimming planes resulting from the CAD integration of the XPV device were used to cut the device model in the Abaqus/Explicit solver (v2021hf7, Dassault Systems, USA), as illustrated in Fig. 2C. A shell-to-solid coupling was defined between the edge surface of the pulmonary wall and the solid boundaries of the resected XPV model. A tie constraint was implemented for the pulmonary wall surface that lies on the inside surface of the XPV device. The general contact algorithm was used to manage interactions between components using hard normal behavior for preventing element penetration. The distal ends of the pulmonary trunk were first extended to a length approximately six times the outlet vessel diameter and then constrained in the longitudinal direction. In a different way, the proximal extension of the right ventricle was fixed in all directions.

### FSI Analysis

**Fig. 3:**
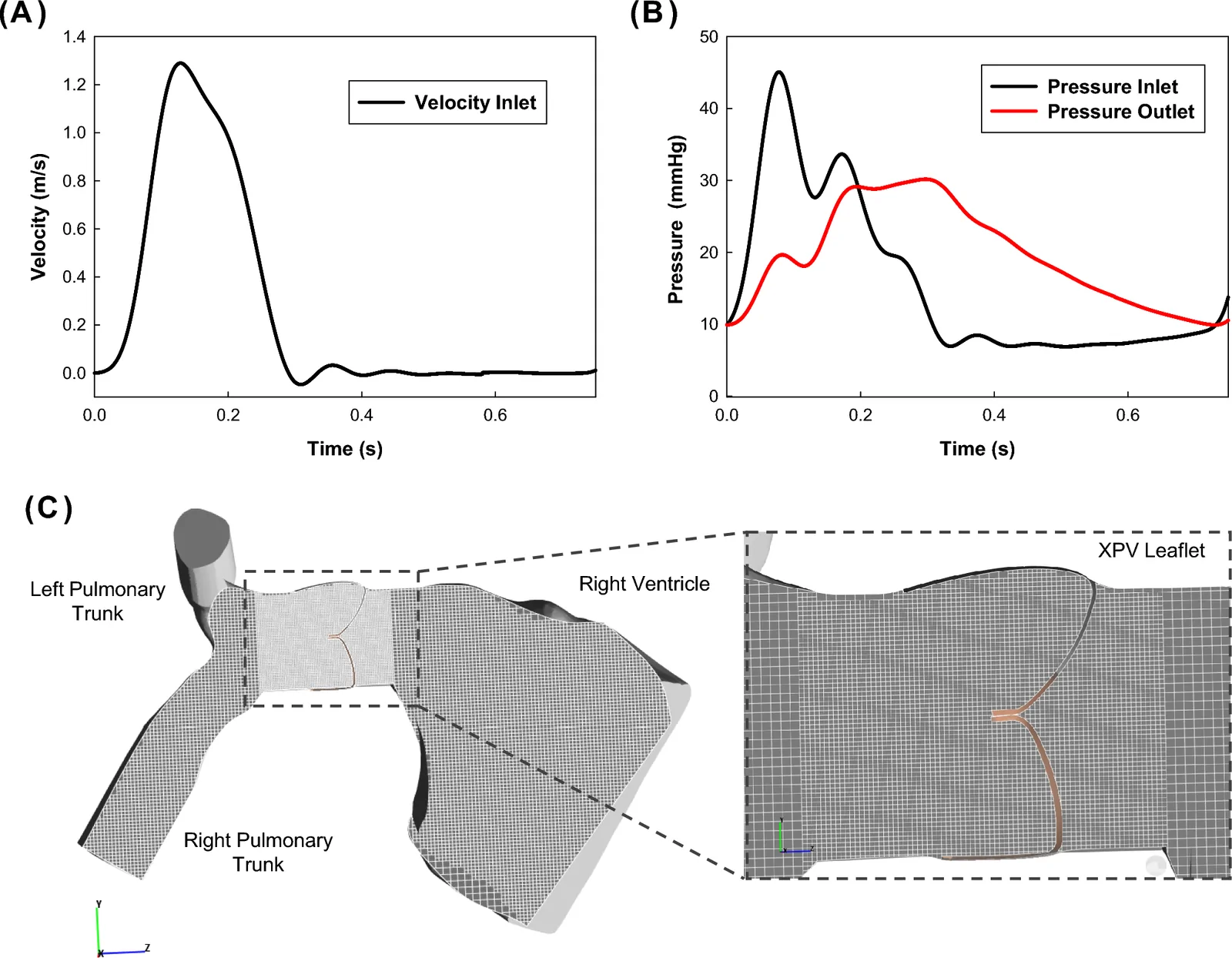
**A** Inflow velocity boundary conditions at the right ventricular inlet and **B** pressure inlet and outlet curves, **C** element grid of the flow solver showing the one-level adaptation in the region of XPV valve leaflets

Flow analysis was carried out using the FlowVision (v3.13, Capvidia, Belgium) software based on a sub-grid geometry resolution approach. The methodology does not necessitate global remeshing at each iteration and relies on a finite-volume-based boundary conforming method. The computational grid was developed from an initial Cartesian grid with a characteristic dimension of 0.6 mm and local static volume adaptations, meaning that the initial grid was refined in the valve leaflets’ region of the fluid domain, and the cell sizes remain constant throughout the simulation (Fig. 3C). The selected mesh size is derived using sensitivity analysis to ensure a discrepancy of less than 3% in the effective orifice area and transmural pressure gradient estimations relative to the coarse mesh assessment. Local quantities such as wall shear stress were evaluated using locally refined grids in the leaflet region to ensure stable estimates. The computational fluid grid consisted of approximately 7.5 × 10^5^ cells after local refinement in the region of the valve leaflets. The sensitivity analysis included the definition of the flexibility parameter and viscous pressure on the luminal surface of each anatomic part.

The fluid was characterized as laminar with Newtonian properties, with a density of 1060 kg/m^3^ and a viscosity of 0.004 Pa·s. The boundary conditions included a velocity inflow during ejection to regulate cardiac output and a pressure inflow during diastole to enhance valve closure (Fig. 3A and B). Equal right and left pulmonary trunk pressure waveforms were set at the bifurcation (Fig. 3B). Velocity and pressure profiles were estimated from literature data [15], while the duration of these profiles was scaled according to the patient’s heartbeat.

Abaqus co-simulation engine was adopted to develop the fully coupled two-way FSI analysis. The luminal surfaces of the pulmonary and XPV walls, as well as the valve leaflets, were adopted for data exchange among solvers using a time step of 1e-4. This value ensured numerical stability of the rapid leaflet dynamics and key quantities of interest during the cardiac cycle and was selected based on preliminary sensitivity analyses. Prior to FSI analysis, a finite-element simulation of the heart cycle was run to pressurize the patient-specific XPV model. Additionally, a VUAMP subroutine was employed for the initial loading of the flow model to prevent any instability. Three cardiac cycles were executed on a server equipped with n.2 AMD EPYC 75F3 (64 CPUs), with 24 parallel processing capabilities for the flow solver and 10 for the structure solver. A total of 12 patient-specific FSI simulations were performed, one for each reconstructed patient anatomy, enabling a cohort-based assessment of the XPV device performance.

### Model Validation

A descriptive approach based on cumulative distribution functions (CDFs) was adopted to assess the agreement between in silico predictions and in vivo measurements. The transmural pressure gradient was selected as the key quantity of interest, as this represents the only quantitative parameter reported in the Xeltis clinical trial [5]. Thus, the CDF represents the probability that the transmural pressure gradient takes a value less than or equal to a given threshold. Agreement between in silico and in vivo results was quantified by calculating the area enclosed between the corresponding CDF curves (referred to as the area metric). The percentage difference between these areas was used as an indicator of the level of agreement in the transmural pressure gradient between model predictions and clinical observations.

## Results

**Fig. 4:**
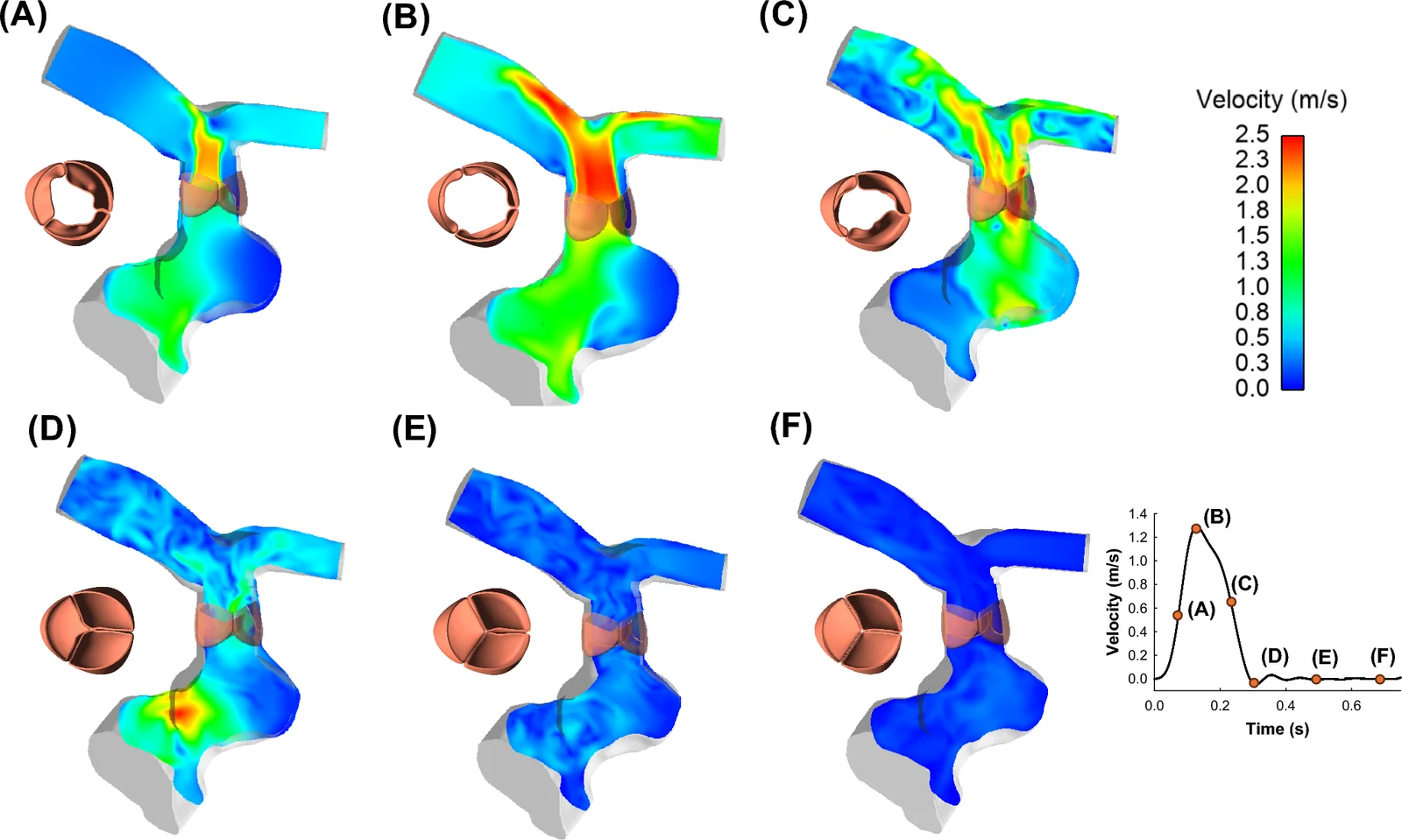
Flow velocity map for a patient-specific XPV model with a size of 16 mm over several steps of the cardiac cycle

For a representative patient-specific XPV model with a size of 16 mm, the flow velocity pattern over the cardiac cycle is highlighted as a colored map together with the device kinematic (Fig. 4). During ejection, the blood flow exhibited a central jet from the valve leaflets impinging the bifurcation of the pulmonary artery. Velocity peaked at 2.45 m/s during systole, then diminished during deceleration, accompanied by a minor backflow before the complete closure of the valve leaflets. During diastole, the FSI model demonstrated its ability to completely seal the right ventricle when a slow-moving flow was present. Detailed results for all patient-specific simulations are available through the public SimInSitu repository (https://siminsitu.eu/repository), enabling full access to the result dataset beyond the representative cases here shown.

Hemodynamic parameters were predicted for each model and displayed in Table 1 as mean and standard deviation. No statistical differences were observed in the predictions of cardiac output, effective orifice area, transmural pressure gradient, and regurgitation fraction across the various sizes of XPV devices.

**Table 1:** Predicted hemodynamic parameters grouped by device size

	XPV		
	16 mm	18 mm	20 mm
CO (L/min)	4.76 ± 0.51	3.97 ± 0.71	3.89 ± 0.84
EOA (cm^2^)	1.21 ± 0.06	1.37 ± 0.13	1.39 ± 0.14
TPG (mmHg)	20.13 ± 0.74	11.65 ± 3.19	14.15 ± 3.07
RF (%)	2.80 ± 1.74	4.41 ± 2.07	4.68 ± 2.35

*CO* cardiac output, *EOA* effective orifice area, *TPG* transmural pressure gradient, *RF* regurgitation fraction

**Fig. 5:**
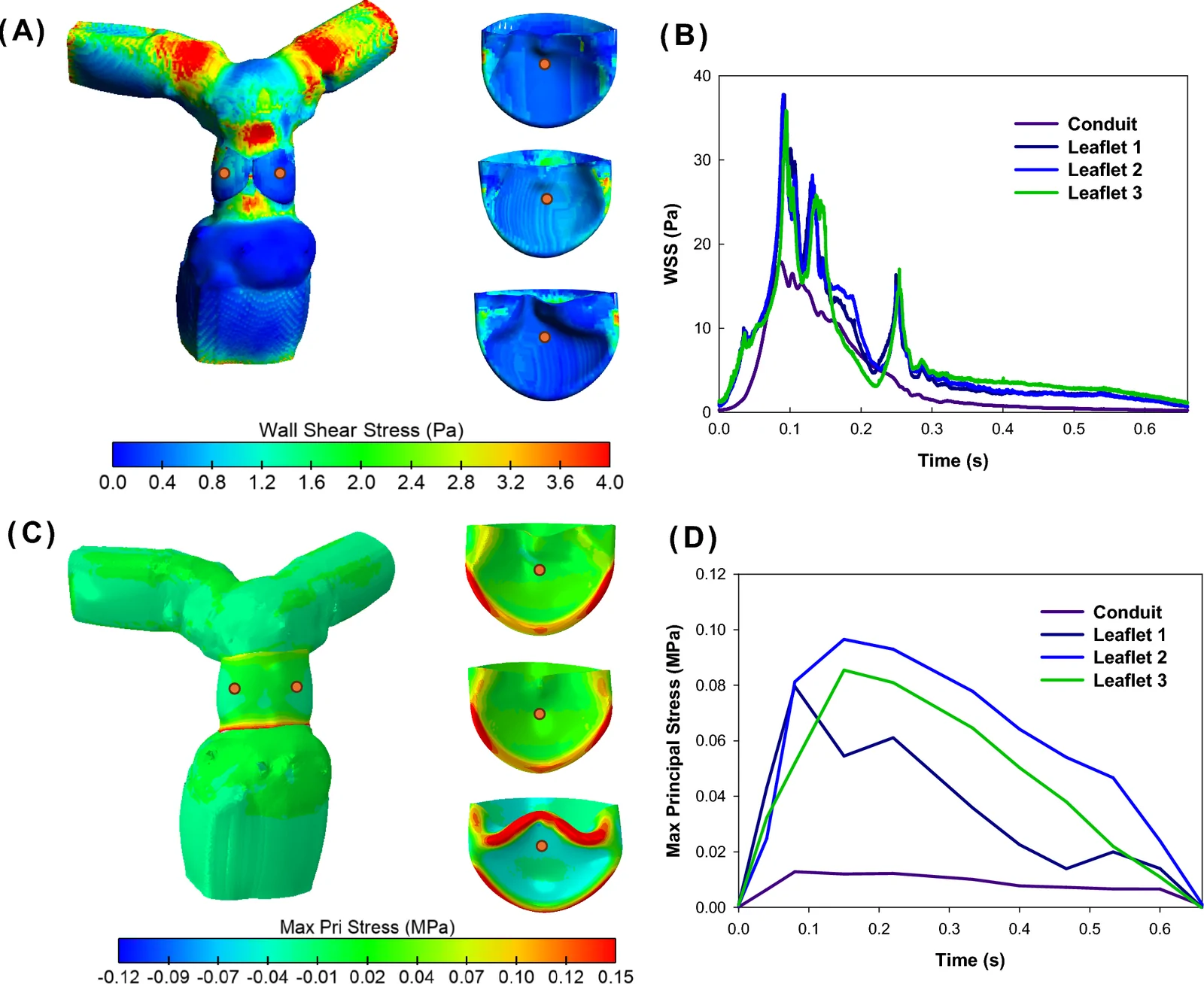
**A** colored map of wall shear stress and **C** maximum principal stress for a patient-specific XPV model with a size of 20 mm; profiles of **B** shear stress and **D** principal stress over the cardiac cycle. Red dots indicate regions for parameter extrapolation

An analysis of the structural and hemodynamic stress of a patient-specific XPV model was done by calculating the wall shear stress and maximal principal stress at the mid-height of the conduit and each valve leaflet (Fig. 5). During peak systole, maximum shear stress was observed at the arterial bifurcation and the distal junction between the XPV device and the pulmonary vessel. The shear stress profile indicated that fluid forces are greater on the luminal surface of valve leaflets compared to the conduit segment. Analogous trends were noted for the maximum principal stress on the XPV device over the cardiac cycle. The pulmonary vessel wall exhibited a homogeneous stress distribution, whereas the valve leaflets displayed stress maxima at the cusp hinges, where the biodegradable material occasionally folds during leaflet opening.

**Fig. 6:**
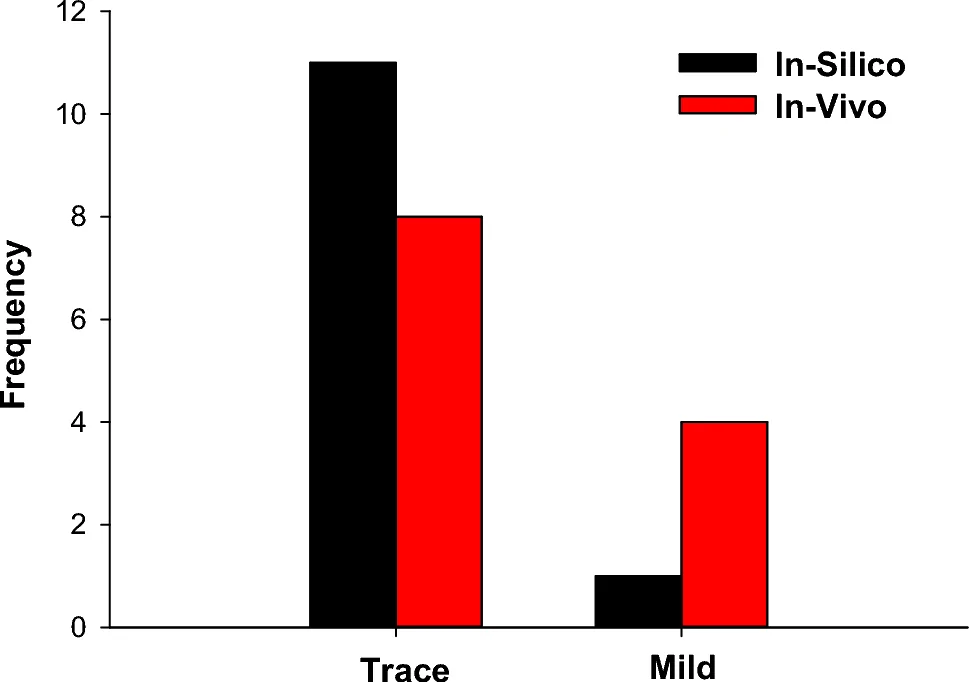
Frequency plot of regurgitation fraction grade among approaches for all patient models

**Fig. 7:**
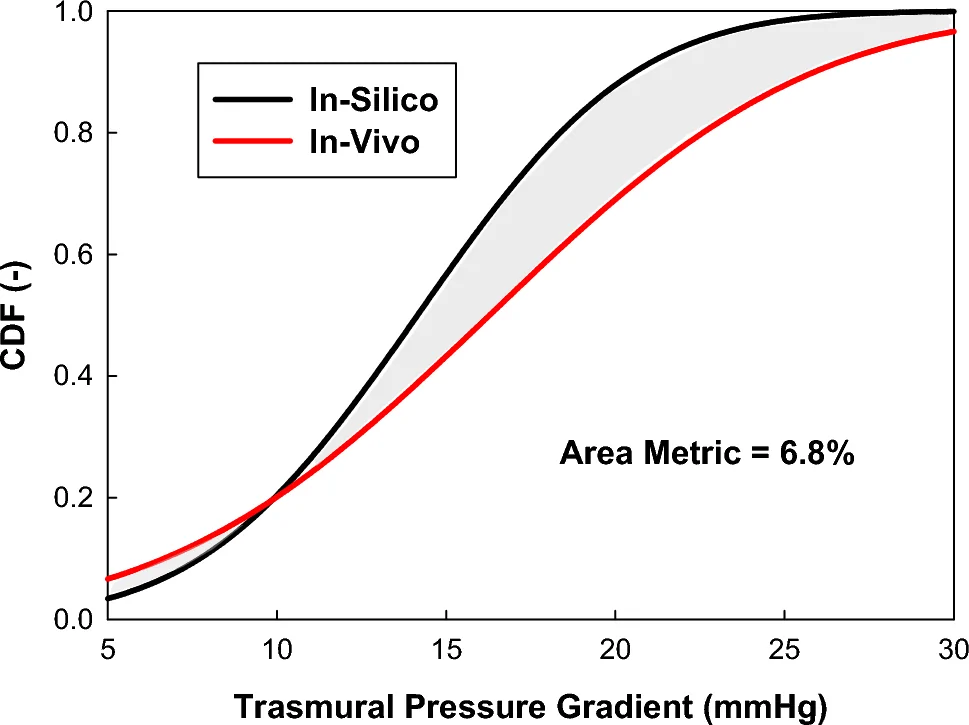
CDF curve between in vivo and in silico showing the area metric (gray region) confined by CDFs

To validate the patient-specific XPV model, a comparison was conducted between the numerically predicted regurgitant volume and the transmural pressure gradient. The regurgitation fraction predictions were initially classified as trace (< 10%), mild (10–20%), moderate (20–40%), and severe (> 40%) [16], afterward represented as a frequency plot in Fig. 6. Simulations demonstrated trace and mild estimates of regurgitation in agreement with echocardiographic assessments, which revealed trace to mild grades at 7 days post-implantation. The discrepancy between in silico and in vivo estimates is likely attributable to differences in the methodological approaches used to quantify regurgitation (i.e., quantitative in silico computation versus echocardiographic assessment). The CDF was calculated for both in vivo and in silico data for the transmural pressure gradient parameter, whereas the area metric was used as a quantitative variable to determine the degree of agreement between the approaches (Fig. 7). An area metric of 6.8% indicated a good concordance between in silico and in vivo assessments of the pressure gradient over the XPV device.

## Discussion

This work utilized in silico modeling to assess the biomechanical response of the Xelits tissue-engineered heart valve when virtually implanted in pediatric patients with congenital heart defects. The most relevant observations and achievements can be summarized as follows:

- A CAD framework was built to replicate the surgical procedure of the XPV device for the reconstruction of the heart valve substitute, as performed in clinical settings. This method enabled the assessment of the device position within the patient pulmonary artery and the assessment of the excessive materials produced by the biofabrication process that need to be cut during surgery. This framework may serve as a pre-operative planning tool to facilitate surgical operations.
- A fully coupled FSI computational model was implemented to characterize the biomechanics of the XPV device using patient-specific anatomies, followed by a comparison of key hemodynamic metrics with retrospective in vivo data. The probabilistic evaluation of transmural pressure gradient indicated a good acceptability of the in silico model relative to in vivo echocardiography. The output is pivotal for assessing the credibility of the proposed patient-specific XPV model.
- An analysis of the fluid shear force and material stress over the cardiac cycle was carried out to determine the role of relevant metrics likely governing the endogenous tissue restoration after implant and determining the scaffold potential for regenerative tissue restoration. The integration of the patient-specific XPV model output with growth and remodeling technique can enable the development of in silico clinical trial to assess the long-term response of the XPV device.

The Xeltis bioresorbable porous polymer valve is engineered for in vivo endogenous tissue restoration, using biomechanical stimuli for growth and remodeling generated by the heart pumping activity in the human host. Cells infiltrate within the porous scaffold and later differentiate into neo-tissue post-implantation. Preclinical investigations of the XPV device in ovine models revealed tissue development on the conduit wall at two months, in addition to functioning leaflets exhibiting vascularization and collagen-rich tissue growth between six and twelve months post-surgery [17]. The first-in-human trial reported by Morales et al. [5] exhibited encouraging clinical results in two cohorts of children, with no requirement for re-intervention after twelve months post-device implantation. In the first study group, significant regurgitation of the Xeltis valve leaflets was noted and then corrected through the redesign of the valve leaflets, as evidenced by the implementation of the second-generation device in another group of six children. This was achieved by the use of computational modeling to determine stress distribution on valve leaflets, hence minimizing areas of stress concentration. Enhancing understanding of the biomechanical performance of this complex device by in silico modeling is essential to accelerate clinical translation. This study established an in silico model to enhance our understanding of the Xeltis device by estimating structural and hemodynamic parameters such as the regurgitation fraction. In all patient models, mild pulmonary valve regurgitation was numerically predicted. This diverged from clinical outcomes revealing cases of moderate and severe regurgitation. We hypothesize that discrepancies could originate from patient bias, flow boundary conditions, and methodological approaches employed in calculating regurgitant volume (i.e., qualitative echocardiographic evaluation versus quantitative in silico assessments). The present computational model allowed the comparison of the predicted transmural pressure gradient with in vivo echocardiographic data 7 days post-surgery. The area metric indicated an acceptable discrepancy (< 10%) between in silico and in vivo results, which is encouraging for establishing the rigor of the proposed FSI modeling technique. This result however fails to broaden the predictive potential of the FSI model for a large patient cohort due to the limited clinical data adopted for comparison and the inclusion of unmatched patients. From a technological standpoint, the FSI model well represents the physiological behavior of the XPV device in the patient body, therefore serving as a comprehensive tool for further validation in the ongoing clinical trial.

Upon implantation, the pulmonary bioresorbable valve experiences significant growth and remodeling as a result of the porous material’s exposure to systemic hemodynamic and structural stresses. The ability to predict how the XPV device enables cell growth and remodeling in the valve structure as a function of the biomechanical properties and patient physiological conditions can enable the evaluation of whether certain patients respond to favorable adaptation and long-term function or maladaptation on the other hand. Middendorp and colleagues [18] performed a computational analysis of pulmonary autograft valve leaflets within the context of the Ross technique, demonstrating that heart valve adaptation can be effectively represented by growth and remodeling principles aimed at tissue repair by mechanical homeostasis. Visser et al. [19] employed finite-element methods for strain analysis of tissue-engineered heart valves to improve the valve geometry by mitigating the area of significant compressive strain observed in the hinge region and valve lunula. Indeed, the optimization of strain distribution was expected to improve long-term physiological performance by promoting the removal of fibrin deposits and preventing blood stagnation. Our FSI model of the XPV device enabled the assessment of patient-specific hemodynamic loads with a level of detail unrivaled by earlier computational analyses. Predictions of shear forces and hydrostatic stress on valve leaflets align with observations from in vitro analysis [20] and simulations of bioprostheses [21]. These parameters can be used as inputs for homogenized constrained mixture theory to determine collagen remodeling, scaffold degradation rates, and anisotropic growth over time. The present FSI model can thus be integrated with a growth and remodeling numerical technique [22] to predict device adaptation in a patient-specific manner, ultimately aiming to account for inter-patient variations that may influence functional remodeling. Future research by our SimInSitu consortium seeks to establish in silico clinical trials to determine the long-term response of the XPV device, utilizing either actual patient models or synthetic cohorts to examine borderline patient anatomies that might lead to device underperformance.

There are several limitations in this study. Blood flow was modeled under laminar flow conditions. Based on characteristic values of peak velocity and vessel diameter, the Reynolds number in the pulmonary artery was estimated to fall within the transitional regime. However, localized turbulent effects may occur near the valve and bifurcation. The absence of a turbulence model may therefore influence local flow features and represent a limitation of the present study. The pressure boundary conditions adopted from literature data may contain low-frequency oscillations associated with experimental measurement setups. While these features are not expected to significantly influence cycle-averaged hemodynamic metrics, they may affect instantaneous flow fields at specific time points. This constraint should be considered when interpreting localized flow features. Furthermore, boundary conditions were derived from literature data and scaled according to patient-specific parameters, including heart rate and cardiac output. This approach enables a partial personalization of the hemodynamic environment, not capturing specific patient pressure and flow waveforms. The acquisition of such data is particularly challenging in pediatric patients with congenital heart disease, even in prospective investigations. This limitation should be considered when interpreting the predicted hemodynamic quantities. A discrepancy was observed between in silico predictions and clinical estimates of regurgitation fraction, and this difference may be partly attributed to the distinct methodological approaches used for the evaluation. Regurgitation is computed quantitatively in the simulations, but qualitatively from echocardiography in clinical practice. Such differences in measurement techniques may introduce variability when comparing the two datasets. Overall, the objective of the present study is not to develop a fully predictive model of post-implantation valve performance, but rather to establish a computational framework for investigating device biomechanics. Therefore, the model should be interpreted for mechanistic insights rather than direct clinical prediction.

## Conclusion

A computational framework was established to first integrate the XPV device into patient-specific anatomies and then evaluate the device’s biomechanical performance using FSI analysis. Upon comparison with in vivo data, the predictive capability of the patient-specific XPV model was found to be acceptable for global hemodynamic metrics, such as the transmural pressure gradient, while discrepancies were observed in the estimation of the regurgitation fraction. Representative flow patterns and stress distributions on the device wall and valve leaflets were obtained, consistent with expected physiological behavior and supported by the agreement observed in global hemodynamic metrics. The current computational framework serves as a comprehensive tool for evaluating tissue-engineered heart valves, aimed at enhancing device design and minimizing the trial and error phase in preclinical studies.

## Data Availability

The datasets generated during and/or analyzed during the current study are not publicly available due to ethical issues but are available from the corresponding author on reasonable request.
